# 869. A Qualitative Review of Social Barriers Impeding Retention in HIV Care at a Ryan White Clinic

**DOI:** 10.1093/ofid/ofab466.1064

**Published:** 2021-12-04

**Authors:** Eleni Florakis, Smith Johanna, Alyssa Kennedy, Lisa A Spacek

**Affiliations:** Thomas Jefferson University, Philadelphia, Pennsylvania

## Abstract

**Background:**

Ending the HIV Epidemic: A Plan for America aims to decrease new HIV diagnoses 75% by 2025 and 90% by 2030. To achieve this, we identified patients unable to achieve viral suppression with social-behavioral needs deemed ‘high-hanging fruit.’ Via extensive outreach efforts and creation of shared problem solving, we pursued the goals of rapid and effective treatment leading to viral suppression and prevention of HIV transmission. We (1) exhausted all avenues of outreach to re-engage patients in HIV care and (2) identified personal or social characteristics related to difficulties in visit retention and achieving viral suppression.

**Methods:**

Of 446 Ryan White-eligible patients seen in an urban, academic medical center, 46 did not achieve and/or maintain viral suppression, and qualified for the study. We conducted a mixed methods survey comprised of both multiple choice and open-ended questions to ascertain what barriers patients face to continuous engagement in care and to achieving viral suppression. We developed a re-engagement outreach cycle which included: text messages and phone calls, electronic messages via patient portal or email, phone call to pharmacy to cross-check contact information, outreach to patients’ emergency contact, and sending a letter by mail.

**Results:**

Of 46 participants, 32 were reached and 14 were not found. Sixteen re-engaged in care and of these, 14 completed the survey (see Figure). Those who completed the survey noted the following barriers to care: poor mental health, financial issues, problems committing to an appointment due to work/family/transportation, and COVID-19. Out of all 46 participants, the 14 who were not found had an overall a higher index of chaos. This index of chaos included, but was not limited to: homelessness, IV drug use, domestic violence, and stigma.

Outreach to re-engage in HIV care

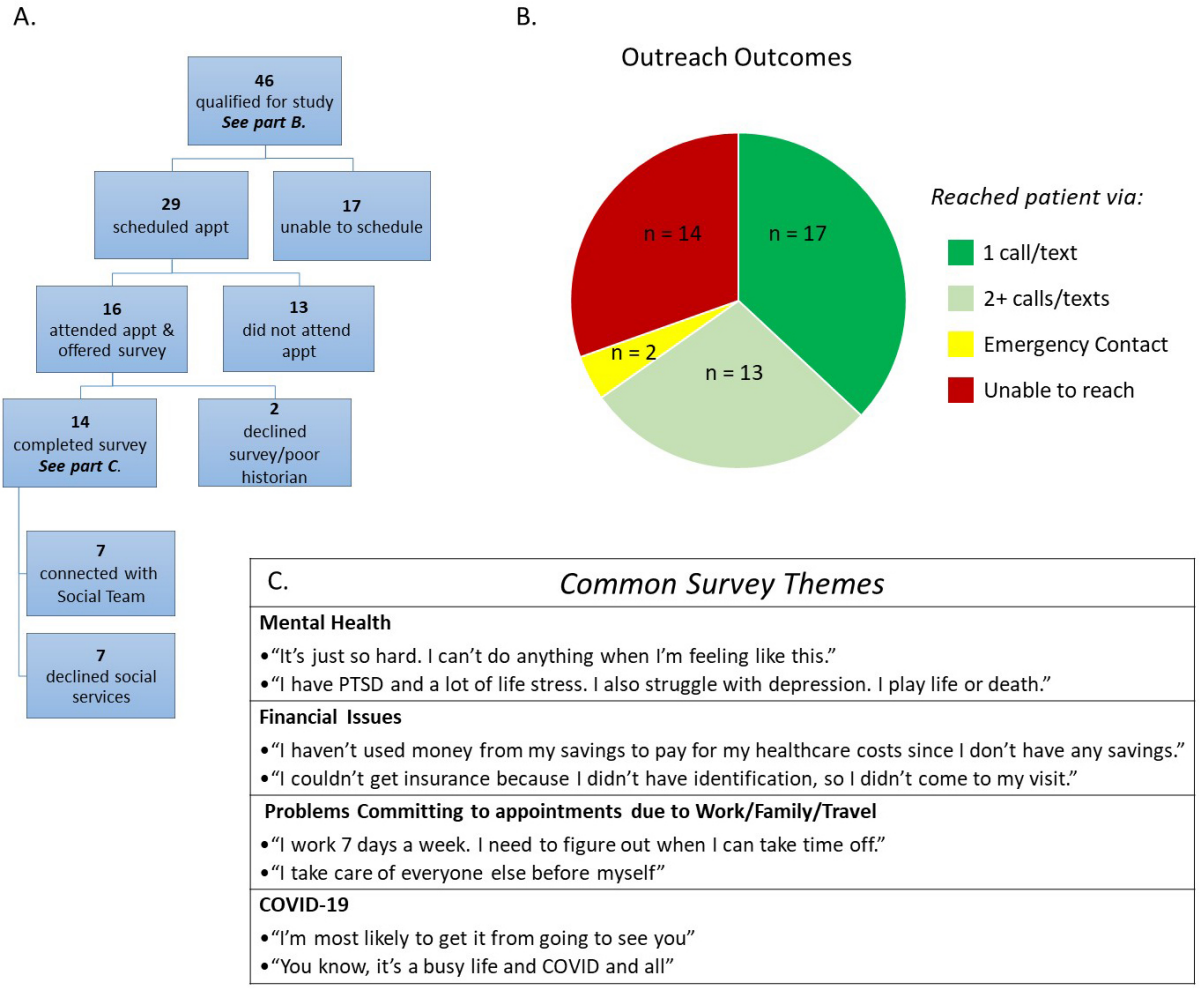

A. Participants in study, B. Outreach outcomes, C. Common survey themes

**Conclusion:**

Intensive efforts are required to re-engage patients, counsel on adherence, and achieve viral suppression. The reasons for lack of engagement in care are real and challenging. Multiple cycles of continuous outreach serve to establish trust, address barriers, and connect to HIV care.

**Disclosures:**

**All Authors**: No reported disclosures

